# ACC-deaminase producing microbial biostimulants for agriculture and climate resilience: barriers and the way forward

**DOI:** 10.3389/fpls.2026.1790494

**Published:** 2026-04-21

**Authors:** Rishu Thakur

**Affiliations:** Menzies School of Health Research, Charles Darwin University, Alice Springs, NT, Australia

**Keywords:** abiotic stress, climate change, fertilizer, plant growth-promoting rhizobacteria (PGPR), soil

## Introduction

**Figure 1:**
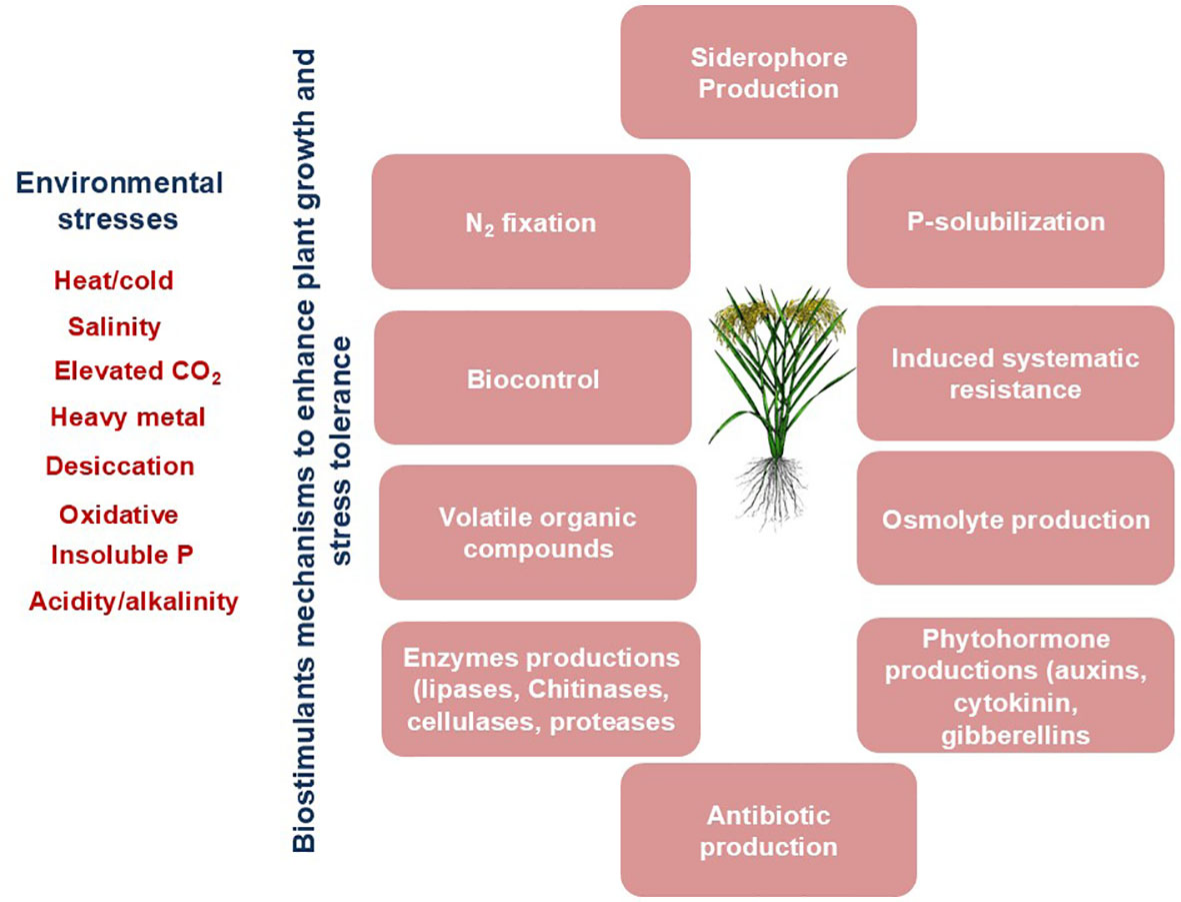
Mechanisms of biostimulant-mediated plant growth promotion and stress tolerance.

Microbial biostimulants (comprising microbes, consortia of microbes, or bioactive compounds) hold immense potential in enhancing plant growth and mitigating the detrimental impacts of environmental stresses (Castiglione et al., 2021) (**Figure 1**; **Table 1**). Soil microbes, particularly 1-aminocyclopropane-1-carboxylate)-deaminase (ACCD)-producing rhizobacteria, regulate plant growth and development under stressed conditions (Glick, 2014; Gulati et al., 2024). ACC is an intermediate precursor in ethylene biosynthesis. Excessive ethylene levels negatively affect root and shoot growth and hamper overall plant performance. These detrimental impacts of ethylene can be overcome by the ACCD enzyme, which can reduce ethylene levels by metabolizing ACC, a precursor of ethylene, and assist in coping with environmental stresses.

**Table 1:** Abiotic stress tolerance by ACC-deaminase producing microbial biostimulants.

Stress	ACC- Deaminase producing microbial biostimulants	Source	Growth promotion response	References
Salinity	*Aneurinibacillus* sp., *Bacillus amyloliquefaciens*, *B. cereus*	Garlic, wheat, cucumber	Increase in dry and fresh, enhanced seed germination, improve plant growth, Increased chlorophyll, antioxidants, and osmolytes.	(Chen et al., 2016; Gupta & Pandey, 2020; Khan et al., 2017)
Drought	*Aneurinibacillus aneurinilyticus*, *Serratia plymuthica*	Garlic, jujube	Enhanced protein and gene expression patterns, increase in dry and fresh weight, shoot biomass, antioxidants and osmolytes.	(Gupta & Pandey, 2019; Zhang et al., 2020)
Heat	*B. cereus*, *Ochrobactrum pseudogrignonense*, *Pseudomonas* sp., and *B. subtilis *	Tomato, mustard, common bean, pea, wild cabbage, and black gram	Promoted shoot and root length, leaf surface area, elevated production of reactive oxygen species scavenging enzymes and cellular osmolytes and higher leaf chlorophyll content.	(Mukhtar et al., 2020; Saikia et al., 2018)
Osmotic stress	*P. aeruginosa* and *B. subtilis*	Leguminous crops	Increased root-shoot length, fresh and dry weight of root and shoot, root-shoot biomass, total sugar, protein, reducing sugar, chlorophyll content, and antioxidants enzymes.	(Gupta et al., 2022)
Heavy metal	*B. gibsonii*, *B. xiamenensis*, *Enterobacter* sp., and *Ochrobactrum* sp.	Sesban, rice	Enhanced antioxidant enzyme activities, amylase and protease activities, increased osmolyes and decreased protease activity	(Mitra et al., 2018; Zainab et al., 2021)

ACCD rhizobacteria have been widely studied as plant growth-promoting rhizobacteria (PGPR) from different crops across the world. Several ACCD PGPR, such as *Bacillus* (Danish and Zafar-ul-Hye, 2019), *Ochrobactrum* and *Pseudomonas* (Saikia et al., 2018), *Acinetobacter*, *Agrobacterium*, *Arthrobacter*, *Cellulomonas*, *Enterobacter*, *Microbacterium*, *Neomicrococcus*, *Priestia*, and *Rhizobium* (Gulati et al., 2024), have been reported to enhance plant growth and abiotic stress tolerance in different crops. Aside from metabolizing ACC, these biostimulants produce organic acids that help in the solubilization of phosphorus, indole acetic acid (IAA)-like auxins that can enhance root growth, and siderophores that enhance iron uptake and its availability to the plants (Thakur et al., 2025).

## Translational gaps in ACCD biostimulants commercialization

Nonetheless, although several ACC-deaminase biostimulants have been identified, characterized, and screened for growth promotion, they have not been commercialized on a large scale. Most studies on microbial biostimulants have been confined to researchers and laboratory conditions. Many factors influence the performance of these biostimulants in real-world settings. Field conditions such as soil pH, moisture, organic matter, and native microbes intensely influence the effectiveness of the biostimulants by affecting microbial survival, colonization, and metabolic activity, which in turn determines their ability to promote plant growth (Azarbad and Junker, 2024). It is also important to note that biostimulants alone do not work effectively in field conditions (Shahwar et al., 2023).

The long-term efficacy of biostimulants under changing climate remains uncertain. Climate change puts pressure on all sectors, particularly those that are dependent on natural resources like agriculture. Changes in climatic conditions affect the soil’s biological, chemical, and physical properties, thus influencing soil health and crop productivity (Oishy et al., 2025). Climate change has worsened catastrophic bushfire events that release carcinogenic compounds into the soil. The agricultural sector is also facing challenges to increase productivity to feed the growing global population. Unsustainable agricultural practices such as the use of chemical fertilizers put pressure on the soil and lead to environmental pollution (De Corato et al., 2024). Only half of these chemical fertilizers are used by the plants, and the remainder is lost to water bodies, causing eutrophication and leaching. Reflecting on the current climate change and unsustainable agriculture practice scenarios, the implementation of sustainable agriculture practices is a must.

## Adoption constraints and strategic solutions

A lack of farmer awareness and disbelief toward biologicals limit adoption, and poor research-industry–government linkages hinder the commercialization of microbial biostimulants (Hasan et al., 2025). Farmers often prioritize inputs that offer short-term returns (Touch et al., 2024). Farmer training with proper demonstration trials and capacity building is needed. Also, farmers’ traditional knowledge should be incorporated into the field application process. Success stories must be recognized and disseminated through local languages at different farmer platforms. The dissemination of both positive and negative outcomes is equally important. Publishing negative results and conducting meta-analyses can also offer insights into context-specific relevance. Regulatory and commercial reforms with clear guidelines are also required. Governments should streamline regulations for microbial biostimulants and recognize them as distinct from chemical pesticides or fertilizers. Governments should also increase collaboration between agribusiness start-ups and researchers. Regional agribusiness hubs that focus on sustainable agriculture solutions and capacity building should be established. Transparent and science-based risk assessment protocols should be developed and then applied. More funding for research and development through grants should be allocated. Transdisciplinary research is also necessary to increase awareness of the role of soil health in human nutrition and health.

Microbial biostimulants must be tested in diverse climatic and soil types. For instance, regions like Punjab and Haryana in India, which are the hub of agriculture and currently facing issues of salinity and low groundwater levels (Krishan et al., 2022; Malik et al., 2023); Sub-Saharan countries including Ethiopia, Kenya, and Nigeria, where drought and low-nutrient soils are major issues (Lombe et al., 2024); and Southeast Asian countries like the Philippines and Vietnam, where natural disasters like typhoons affect rice production (Yuen et al., 2024), should be selected to evaluate the efficacy of native biostimulants. Countries like Australia, which has seen a shift regarding extreme temperatures and lower rainfall (CSIRO, 2024), can be an important site to evaluate biostimulants’ efficacy. Climate-sensitive crops such as maize and rice should be prioritized for multi-location field testing.

ACCD alone may not be an effective strategy. It has been well documented that biostimulants with multiple plant growth-promoting activities are more effective in enhancing plant growth, stress tolerance, and overall productivity than a singular focus on one PGP trait (Gulati et al., 2024). Co-selection for other traits like biotic stress tolerance, nitrogen fixation, phosphate solubilization, and siderophore production can improve efficacy and consistency (Thakur et al., 2024). The use of consortia, biochar, arbuscular mycorrhizal fungi, and other organic amendments can be useful to enhance their performance under field conditions.

In the future, tools such as smart sensors and AI-based decision support systems may also play a role in guiding the application of microbial biostimulants in agriculture. Smart sensors provide real-time data on soil moisture, temperature, salinity, and overall plant health, which allow early identification of stress conditions (Pace et al., 2025). AI-based decision support systems integrate these data with weather forecasts and crop growth models to refine strain selection as well as the timing and dosage of inoculation (Rezaee Danesh, 2025). This integrated approach can improve biostimulant survival, root colonization, and overall stress-alleviation performance under field conditions. Omics approaches such as transcriptomics and metabolomics can further provide insights into the biostimulants’ interaction with plants under field conditions.
